# Balancing the Treatment of Kaposi Sarcoma and Bullous Pemphigoid: A Therapeutic Challenge in a 63-Year-Old Male

**DOI:** 10.7759/cureus.77708

**Published:** 2025-01-20

**Authors:** Amritpal Kooner, Michelle R Anthony, Dhruv Gandhi, Aditya Dutt, Conor Dolehide

**Affiliations:** 1 Dermatology, Midwestern University Chicago College of Osteopathic Medicine, Downers Grove, USA; 2 Medicine, University of Arizona College of Medicine, Tucson, USA; 3 Medicine, K. J. Somaiya Medical College and Research Centre, Mumbai, IND

**Keywords:** bullous pemphigoid, clobetasol, hiv, imiquimod, immune modulation, immunosuppression, kaposi sarcoma

## Abstract

Kaposi sarcoma is a malignancy evolving from the lining of blood and lymphatic vessels. It is caused by the reactivation of human herpesvirus 8, often due to underlying immunosuppression. Classic Kaposi sarcoma occurs in Eastern European males greater than 50 years of age as painless violaceous papules to nodules. We report a case of a 63-year-old man with severe Kaposi sarcoma first diagnosed on the right medial foot with progressive involvement of upper and lower extremities. It was managed with monthly rounds of excisional cauterization, electrodesiccation, cryotherapy, and 5% imiquimod topical cream, leading to overall improvement and avoidance of amputation. However, a year later the patient presented with bullous pemphigoid on the left distal upper arm, suprapubic skin, and right and left pretibial regions. After three months of treatment with mycophenolate mofetil, prednisone, triamcinolone, doxycycline, and clobetasol, the patient’s bullous pemphigoid stabilized. Hence, the simultaneous presentation of bullous pemphigoid and Kaposi sarcoma displays a unique problem in the management and treatment of dermatological conditions. Kaposi sarcoma is exacerbated through immunosuppression, whereas bullous pemphigoid treatment requires immunosuppressants. Extensive excisional therapy and cryotherapy for Kaposi sarcoma, alongside careful management of immunosuppressants for bullous pemphigoid, is critical for managing optimal care in concurrent cases. Further research should be performed to better understand the causal interplay of Kaposi sarcoma, imiquimod, bullous pemphigoid, and other dermatological issues requiring immunosuppressant treatment.

## Introduction

Kaposi sarcoma (KS) is a vascular neoplasm resulting from herpesvirus/human herpesvirus 8 (KSHV/HHV8) infection. It can affect the skin, mucosa, and viscera, and was first described in 1872 [1,2]. KS is characterized by the proliferation of spindle-shaped cells, erythrocyte extravasation, and neoangiogenesis which results in a highly heterogeneous clinical presentation that poses diagnostic and therapeutic challenges [2,3]. There are presently four distinct types of KS, namely classic, endemic or African, AIDS-related, and iatrogenic KS [4]. KSHV/HHV8 has the ability to remain dormant in B cells that are positive for CD-19 and is typically transmitted through sexual contact and saliva [3]. The prevailing theory of KS pathogenesis involves cytokine-mediated cellular function disruption, leading to increased proliferation and differentiation of cellular function [5]. Classic KS predominantly affects older men from Eastern Europe or the Mediterranean, presenting as painless violet nodules or papules on the lower extremity, with possible involvement of the lymphatics or gastrointestinal system [3,6]. Endemic KS is common in sub-Saharan Africa and can affect both children and adults, with some cases showing aggressive lymph nodes and visceral involvement [7]. AIDS-related KS is an AIDS-defining illness that presents itself when CD4 counts fall below 200 cells/mm³ and involves cutaneous and internal organ sites [3]. Iatrogenic KS has been shown to occur in immunosuppressed patients such as those who underwent organ transplants, and resolution can be achieved with a reduction in immunosuppression [3].

Concurrent presentation of KS and other immune-mediated dermatological conditions, such as bullous pemphigoid (BP) are rare and pose significant challenges in management. BP is a chronic autoimmune condition that is characterized by subepidermal blister formation due to autoantibodies targeting hemidesmosomes, particularly BP180 and BP230 proteins [8]. This co-occurrence requires careful management to balance and address both the malignancy and autoimmune processes present in these conditions, respectively. This case report highlights the complex interplay between KS and BP, focusing on clinical challenges in diagnosis and treatment and exploring possible treatment modalities.

## Case presentation

Initial diagnosis and background

We present a case study involving a 63-year-old male with severe KS, who experienced BP following a course of excisional cauterization, electrodesiccation, cryotherapy, and 5% imiquimod topical cream. Successfully treating KS, a condition that stems from underlying immunosuppression, poses a distinctive obstacle when formulating a dermatological solution that necessitates immunosuppression.

**Figure 1:**
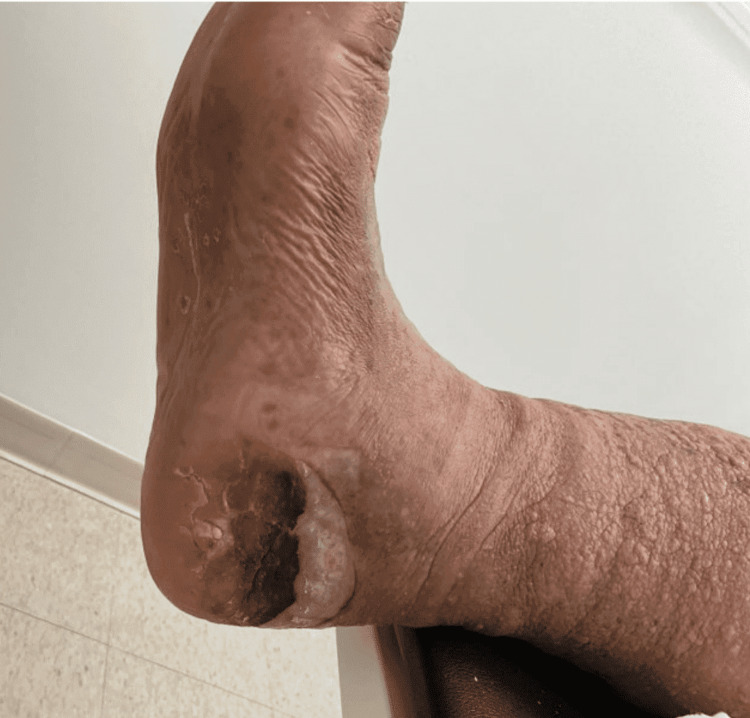
Kaposi sarcoma lesions on right planter mid heel Characteristic violaceous lesions of Kaposi sarcoma were located on the patient's right mid heel, observed at the seven-month follow-up visit. The distinct color and location in weight-bearing areas suggest progression due to repeated trauma and inflammation.

A 63-year-old male, with a past medical history of atrial fibrillation, chronic kidney disease, diabetes mellitus, essential hypertension, gout, and KS-associated herpesvirus inflammatory cytokine syndrome, presented for follow-up treatment for KS, which had initially manifested as violaceous nodules on the right instep and right medial plantar heel (Figure 1). Additionally, the patient had associated elephantiasis nostras verrucosa on both the right and left distal pretibial regions. A punch biopsy of the medial plantar midfoot on the right and a shave biopsy on the medial plantar heel on the same side confirmed the clinical suspicion of KS. Over 18 months, the patient underwent regular monthly cryotherapy sessions with liquid nitrogen and three rounds of excisional cauterization of their right toe. This combination therapy prevented the progression of KS and avoided amputation. The patient was also prescribed daily 5% imiquimod for the management of their KS. The patient’s additional medications include amoxicillin-pot clavulanate, apixaban, atorvastatin, losartan, insulin glargine, albuterol sulfate, amiodarone, gabapentin, hydralazine, hydroxyzine HCl, levothyroxine, carvedilol, allopurinol, atorvastatin, and furosemide. The patient indicates previous alcohol and tobacco use but denied illicit drug use.

Treatment progression

**Figure 2:**
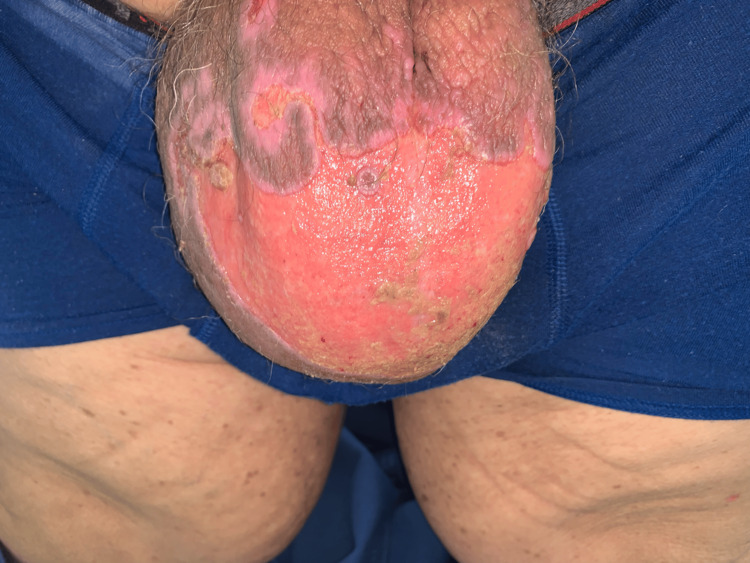
Bullous pemphigoid blisters on the ventral scrotum Ruptured blisters characteristic of bullous pemphigoid located on the ventral aspect of the scrotum. These lesions developed 18 months after initial assessment and led to the initiation of immunosuppression with mycophenolate mofetil and prednisone, which could exacerbate the patient's Kaposi sarcoma.

After 18 months, the patient’s KS improved significantly with a notable reduction in lesions. However, they presented with BP as tense fluid-filled blisters on their left anterior distal upper arm, suprapubic skin, right proximal pretibial region, and left distal pretibial region (Figure 2). Ruptured blisters on the ventral scrotum were also observed. The patient was prescribed twice daily 250 mg mycophenolate mofetil and twice daily 10 mg prednisone. Approximately 20 months after their initial assessment, the patient’s BP had resolved; however, there was a minor outbreak of KS, with violaceous nodules reappearing on the right medial foot and left lateral foot.

Recurrence and long-term management

At the patient’s seven-month, 12-month, and 14-month visits, the patient presented with new painful lesions of Kaposi sarcoma on bilateral elbows. Biopsy by punch method on the right posterior arm confirmed the recurrence of KS. Twelve months after the initial assessment, the patient presented with KS on the left plantar forefoot overlying the first metatarsal. A shave biopsy confirmed the recurrence of KS. Fourteen months following the initial assessment, the patient presented with KS on the right lateral plantar fourth toe, right medial plantar midfoot, and right plantar forefoot overlying the third metatarsal. After each visit, the patient was prescribed 5% imiquimod cream and cryo-destruction with liquid nitrogen. Imiquimod and cryotherapy showed promising results in reducing the progression of existing KS lesions; however, it was less efficient in preventing new KS lesions.

At the 20-month visit, mycophenolate mofetil and prednisone were stopped after two months of use and a daily regimen of head-to-toe clobetasol propionate 0.5% topical ointment was prescribed, alongside an increased frequency of cryotherapy. This regimen was followed for the next six months. Approximately 26 months after the patient's initial visit, and six months after the application of head-to-toe clobetasol propionate and weekly cryotherapy, the patient had significantly reduced BP, and their KS was also controlled. The patient reported an improved quality of life but needed weekly check-ups to prevent the recurrence of both conditions. The full course of the patient's timeline, including treatments, medications, and outcomes, is listed in Table 1.

**Table 1:** Timeline of treatments, medications, and outcomes This table summarizes the chronological progression of the patient's conditions, the treatments administered, and their respective outcomes in the management of this patient's Kaposi sarcoma (KS) and bullous pemphigoid (BP).

Timepoint	Event/Condition	Treatment	Outcome
Initial Assessment	KS on right instep and heel	Cryotherapy, excisional cauterization, 5% imiquimod cream	Significant improvement in KS; prevention of amputation.
7 months	Recurrence of KS on elbows	Cryotherapy and 5% imiquimod cream	Reduced lesion size but incomplete prevention of new lesions.
18 months	BP development on limbs, torso, and scrotum	Mycophenolate mofetil, prednisone	BP resolved within 2 months, but minor recurrence of KS noted.
20 months	Minor KS recurrence	Cryotherapy, clobetasol propionate	KS controlled; significant reduction in BP lesions.
26 months	Follow-up	Maintenance with clobetasol and cryotherapy	Both KS and BP well-managed; improved quality of life but continued need for regular check-ups.

## Discussion

Imiquimod is an imidazoquinoline amine that exerts its pharmacological effects through the activation of toll-like receptors (TLRs) 7 and 8 on macrophages. This interaction leads to the production of interferon-alpha (IFN-α), interferon-beta (IFN-β), and a T-helper type 1 cell-mediated immune response, which plays a crucial role in the pathogenesis of various diseases [9]. The ability of imiquimod to modulate the immune system has made it a promising therapeutic agent for the treatment of viral infections, skin cancers, and other immune-related disorders [10]. Imiquimod, a 5% cream, is used for various cutaneous lesions, including genital warts, actinic keratosis, and superficial basal cell carcinoma [11,12,13]. Celestin et al. conducted an open-label clinical trial on 17 patients with classic or endemic KS treated with imiquimod 5% cream for 24 weeks. The results showed an overall clinical response in 47% of patients. However, the most commonly reported adverse events were erythema and pruritus [14]. These findings suggest that imiquimod 5% cream may be a potential therapeutic option for KS, but further research is required to evaluate its efficacy and safety in larger patient populations [3,14].

Rosen et al. described a case where a patient presented with AIDS-related KS on both lower extremities. After being treated with imiquimod 5% cream and 50 mg/day etoposide for a year, the patient achieved a full recovery. However, they did experience adverse effects such as flu-like symptoms and pruritus that were remedied through dosage adjustments [15]. Similarly, Goiriz et al. reported a case of an 87-year-old man who presented with asymptomatic erythematous-violaceous papules and nodules of two months duration, which were subsequently biopsied, and a diagnosis of KS was made [16]. Oncological treatment with etoposide 50 mg/day in 10-day cycles led to severe side effects from the treatment. This patient was then started on topical 5% imiquimod three times a week for three months, which led to complete resolution [16]. Bernardini et al. presented a case of a 77-year-old Caucasian male treated with 5% topical imiquimod with histologically proven KS, that was later shown to be HIV-positive. This patient was prescribed treatment three times a week for three months, resulting in almost complete clearance of lesions with no local or systemic side effects [17]. These cases represent classical presentations of KS and demonstrate the effectiveness of standard treatments, such as topical 5% imiquimod and etoposide, in the absence of conflicting comorbidities.

In contrast, Li et al. presented a case report of a 72-year-old male suffering from actinic keratoses on the scalp, who developed BP after treatment with 3.5% imiquimod cream. The BP affected the patient's scalp, limbs, and torso [18]. Li et al. posited that the development of BP may be attributed to the immunomodulatory effects of imiquimod, which could lead to a shift in the immune response that is necessary for the pathogenesis of BP [18]. This finding raises concern about the potential for imiquimod to increase the risk of BP in patients with underlying dermatological conditions. Though their case involved actinic keratosis, the case we present describes imiquimod-induced BP in an HIV-negative patient with KS, highlighting a concerning adverse effect in an individual with a disrupted immune response.

## Conclusions

Managing KS and BP concurrently presents a unique clinical challenge due to the opposing treatment strategies required for each condition. Immunosuppression with systemic steroids, essential for controlling BP, can promote the progression of KS, whereas immunomodulatory therapies like imiquimod can lead to adverse side effects such as BP. In this case, opting for head-to-toe clobetasol and increased frequency of cryotherapy was pivotal in managing the patient's concurrent conditions. Unlike systemic immunosuppressants, topical clobetasol provides a localized treatment to prevent exacerbation of opposing conditions. This approach underscores the importance of personalized medicine, focusing on minimizing complications and improving the quality of life for patients. 
